# Comparative metatranscriptome analysis revealed broad response of microbial communities in two soil types, agriculture versus organic soil

**DOI:** 10.1186/s43141-019-0006-3

**Published:** 2019-10-14

**Authors:** Pushpender Kumar Sharma, Vinay Sharma, Shailesh Sharma, Garima Bhatia, Kashmir Singh, Rohit Sharma

**Affiliations:** 1 Sri Guru Granth Sahib World University, Fatehgarh Sahib, Punjab 140407 India; 2 National Institute of Animal Biotechnology (NIAB), Miyapur, Hyderabad, Telangana 500 049 India; 3 Department of Biotechnology, Panjab University, Chandigarh, 160014 India

**Keywords:** Metatranscriptomics, Differential expression, Sequencing, Pollutants, Cypermethrin, Heavy metals

## Abstract

**Background:**

Studying expression of genes by direct sequencing and analysis of metatranscriptomes at a particular time and space can disclose structural and functional insights about microbial communities. The present study reports comparative analysis of metatranscriptome from two distinct soil ecosystems referred as M1 (agriculture soil) and O1 (organic soil).

**Results:**

Analysis of sequencing reads revealed *Proteobacteria* as major dominant phyla in both soil types. The order of the top 3 abundant phyla in M1 sample was *Proteobacteria* > *Ascomycota* > *Firmicutes*, whereas in sample O1, the order was *Proteobacteria* > *Cyanobacteria* > *Actinobacteria*. Analysis of differentially expressed genes demonstrated high expression of transcripts related to copper-binding proteins, proteins involved in electron carrier activity, DNA integration, endonuclease activity, MFS transportation, and other uncharacterized proteins in M1 compared to O1. Of the particular interests, several transcripts related to nitrification, ammonification, stress response, and alternate carbon fixation pathways were highly expressed in M1. In-depth analysis of the sequencing data revealed that transcripts of archaeal origin had high expression in M1 compared to O1 indicating the active role of *Archaea* in metal- and pesticide-contaminated environment. In addition, transcripts encoding 4-hydroxyphenylpyruvate dioxygenase, glyoxalase/bleomycin resistance protein/dioxygenase, metapyrocatechase, and ring hydroxylating dioxygenases of aromatic hydrocarbon degradation pathways had high expression in M1. Altogether, this study provided important insights about the transcripts and pathways upregulating in the presence of pesticides and herbicides.

**Conclusion:**

Altogether, this study claims a high expression of microbial transcripts in two ecosystems with a wide range of functions. It further provided clue about several molecular markers which could be a strong indicator of metal and pesticide contamination in soils. Interestingly, our study revealed that *Archaea* are playing a significant role in nitrification process as compared to bacteria in metal- and pesticide-contaminated soil. In particular, high expression of transcripts related to aromatic hydrocarbon degradation in M1 soil indicates their important role in biodegradation of pollutants, and therefore, further investigation is needed.

## Backgrounds

Understanding microbial community structure and function of the soil ecosystem is vital to delineate ecological roles of the associated microbiome [1, 2]. Several studies have shown that more than 97% of microbes in different ecological habitats (soil, water, acid mine drainage, hot spring) cannot be cultured, and hence, their functional and ecological roles in various biochemical processes remained unexplored [3, 4]. Advances in next-generation sequencing have provided novel insights about structural and functional organization of bacterial genomes and about the key physiological processes and mechanisms bacteria employ to acclimatize under a set of environmental conditions [5–7]. Recently, genome sequencing and analysis of lignocelluloses degrading saprophytic fungi deciphered complete information about the enzymatic machinery these fungi have used to degrade lignocelluloses [8]. Though whole metagenome sequencing and assembly offers opportunity for researchers to profile gene diversity and function under normal conditions, it cannot predict gene functions and pathways which upregulate under particular environmental conditions [9]. In sharp contrast to this, metatranscriptome sequencing and analysis hold immense potential to unravel the functional role of diverse microbiota under various environmental conditions [10, 11]. Nonetheless, next-generation sequencing carried out from couple of complex microbial communities (marine, sediments, and soil) has successfully addressed the role of microbes in various ecological processes [12–17]. In agricultural lands where microbes interact within and between, various groups of organisms makes it very difficult to discover the role of pesticides and metals in shaping microbial community structure and function [18]. Furthermore, large variation in sorption, desorption, and degradation of pesticides has been reported in different soil types [19, 20]. Pesticide usage in agriculture increases the number of pesticides degrading bacteria or archaea in soil [21]. Interestingly, nitrification test as a pesticide side effect has been reported as the best way to depict the role of microorganisms in soil [22]. Studying expression of genes by direct sequencing and analysis of metatranscriptomes at a particular time and space can disclose structural and functional divergence of microbial communities [23].

Spraying of chemicals, e.g., pesticides, herbicides, and fertilizers, in agricultural lands of Punjab (India) is a regular practice [24]. We hypothesized that long usage of such chemicals may alter microbial community structure and function; thus, comparative metatranscriptome can allow researchers to investigate the ecological roles of microbial communities in such environments. Herein, we sequence, analyze, and compare the whole metatranscriptome of agricultural versus organic soil.

## Methods

### Site description and sampling

Soil samples were collected in duplicates from the agricultural field (M1) of Malwa region of Punjab, India (29° 30′ and 31° 10′ north latitudes and 73° 50′ and 76° 50′ east longitudes), and from the normal organic soil (O1) where no modern agricultural practices are carried out. Samples were collected at a depth of 0–10 cm in September 2014 (atmospheric temperature 28 °C in RNA later (Ambion) (2 ml RNA later added to 2 g of soil) and stored at − 80 °C. Agricultural soil was loamy and had pH of 7.5 to 8.0 whereas the organic soil was acidic and had pH of 6.5–7.0; it was deep brown and porous in nature. Soil samples were transported to the laboratory in dry ice and stored in − 80 °C freezer till its further use for extraction of RNA. The estimation of metals in soil was performed by inductively coupled plasma mass spectrometer (ICP-MS Agilent 7700), whereas the pesticides in the two soil types were estimated by gas chromatography-mass spectrometry (GC-MS, Agilent technologies) as reported in our previous study [25].

### RNA extraction

Total RNA was extracted from 2 g soil in duplicates each from M1 and O1 using RNA PowerSoil® Total RNA isolation kit (MO BIO Laboratories, Inc., Carlsbad, CA 92010, USA). Briefly, ~ 2 g soil was homogenized in a 15-ml tube containing silica carbide beads, lysis buffers, phenol to chloroform to isoamyl alcohol (pH 6.5–8), and IRS, to ensure complete lysis of all microorganisms and neutralization of RNases. Clear lysates were precipitated to concentrate the total nucleic acids and were re-suspended in a buffer optimized for binding to anion-exchange gravity flow columns. RNA was eluted using a high salt buffer and was precipitated to obtain the final pure RNA which was resuspended in RNase-free water. RNA from two extractions was pooled (both from M1 and O1) before cDNA preparation and sequencing.

### cDNA library construction and Illumina sequencing

Total RNA was treated with DNase to remove DNA contamination. RNA quality was assessed using NANODROP LITE spectrophotometer (Thermo Fisher Scientific, Waltham, MA, USA). Double-stranded cDNA was generated from amplified RNA using the superscript IITM double-strand cDNA synthesis kit (Thermo Fisher Scientific, Waltham, MA, USA), as per manufacturer’s instructions. Library preparation, processing, and sequencing were performed at the SciGenom Labs Private Limited, Kerala, India, using the Illumina HighSeq2500 with paired-end (PE) sequencing. Data was submitted with MG-RAST server [26]. MG-Rast ID of the data sets is available for O1 with accession number is mgm4653349.3; M1 dataset was provided a Gold ID 0eeec568676d676d343733323034392e33 and is under progress at present; however, it can be accessed upon request to the server.

### FASTQ files quality checking

Raw sequences obtained after the sequencing of metatranscriptomes were analyzed using FastQC tool. Base distribution, base composition, and GC distribution of reads were calculated from the QC result. Based on results, we trimmed the sequence read wherever it was necessary in order to retain high-quality sequences for further analysis. Additionally, reads with more than 10% of “N”s, Illumina adapter contaminated reads, and low-quality reads were excluded from the analysis.

### Adapter removal

Raw sample reads were initially pre-processed by removing adapter (Illumina) sequences. Adapter removal was carried out using the cutadapt tool (version 1.7.1) with default parameters.

### Other non-coding RNA removals

The other non-coding reads such as tRNA and rRNA sequences were filtered via aligning the adapter filtered reads against reference tRNA sequences downloaded from Genomic tRNA database (GtRNA: http://gtrnadb.ucsc.edu/) using Bowtie-2 tool (http://bowtie-bio.sourceforge.net/bowtie2/index.shtml). rRNA sequences were removed using SortMeRNA (http://bioinfo.lifl.fr/RNA/sortmerna/).

### Sequence clustering

The reads from each sample were clustered using UCLUST tool (http://www.drive5.com/usearch) with the cutoff value of > 90%. From clustered sequences, the representative sequencing reads were used as a query for functional annotation.

### Taxonomy and functional annotation of sequencing reads

Read-based annotations of unique cDNA sequences were carried out against non-redundant sequence database using standalone BlastX program (http://www.ncbi.nlm.nih.gov/) with optimal *e* value of 10e−5. The best hits showing sequence similarity greater than 90 and lowest *e* value were retrieved. The predicted gene functions from each read were annotated using Gene Ontology (GO) and KEGG (Kyoto Encyclopedia of Genes and Genomes analysis). The GI (sequence accession number) of each functional hit was retrieved and queried against UniProt database (http://www.UniProt.org/). The transcript id and gene function is provided in the supplementary figures and tables (Additional files 1, 2, 3, 4 and 5).

### Differential expression studies

The analysis of differentially expressed genes was carried out based on the read counts of all the samples using DESeq package (http://bioconductor.org/packages/release/bioc/html/ DESeq.html). Initially, the read counts of common transcripts from all samples were considered as an input table for DESeq. All the transcript frequencies were brought to the common scale to make them comparable by normalizing the read counts from each sample. Differential expression was carried out by negative binomial test, and as a result, the mean read count of sample combination, fold change, and *p* values were estimated. The *p* value between sample combinations shows the significance of differential expression.

## Results

### Soil characterization and estimation of pesticides and heavy metals

The soil surface was sandy loam in all samples, and the pH of soil for the organic and agriculture soil was 8 and 9.2 respectively, at the time of collection. Out of several pesticides analyzed, M1 soil revealed the presence of cypermethrin I and III in the range of 0.019 ± 0.001 ppb, whereas cypermethrin II and IV were found to be 0.018 ± 0.002 and 0.017 ± 0.002 ppb, respectively. Heavy metals such as nickel (47.7 ± 2.91 mg kg^−1^ and 58.7 ± 2.91 mg kg^−1^), mercury (23.8 ± 5.58 mg kg^−1^ and 28.8 ± 5.58 mg kg^−1^), selenium (21.1 ± 4.25 mg kg^−1^ and 21.1 ± 4.25 mg kg^−1^), and cadmium (3.1 ± 0.8 mg kg^−1^ and 5.1 ± 0.8 mg kg^−1^) are also reported in our previous studies [25]. No residues of pesticides and metals were detected in the O1 soil sample.

### RNA extraction, quality checking and, Gene Ontology (GO)

**Fig. 1:**
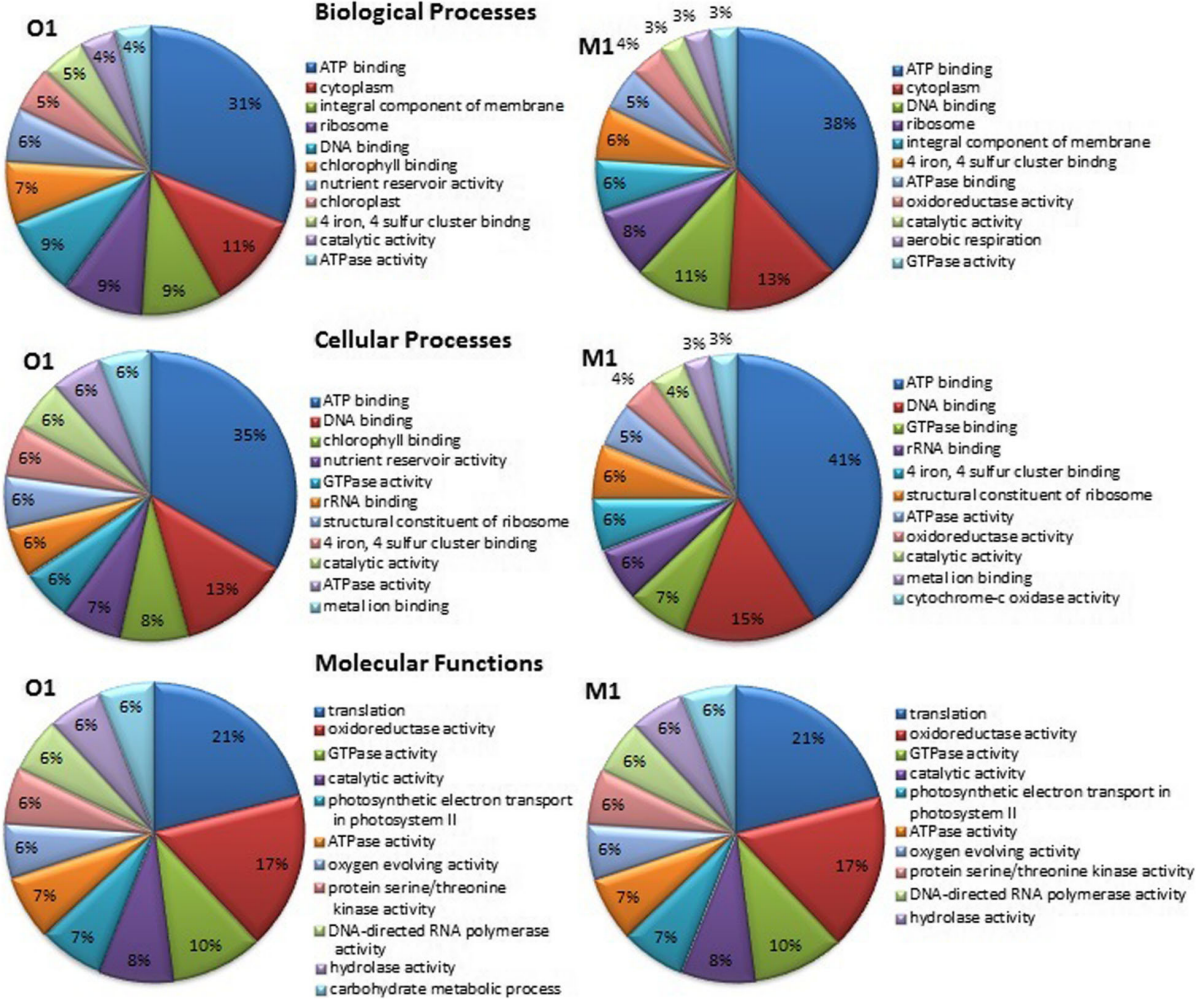
Percent distribution of transcripts assigned to biological processes, molecular functions, and cellular components mapped using Gene Ontology

**Fig. 2:**
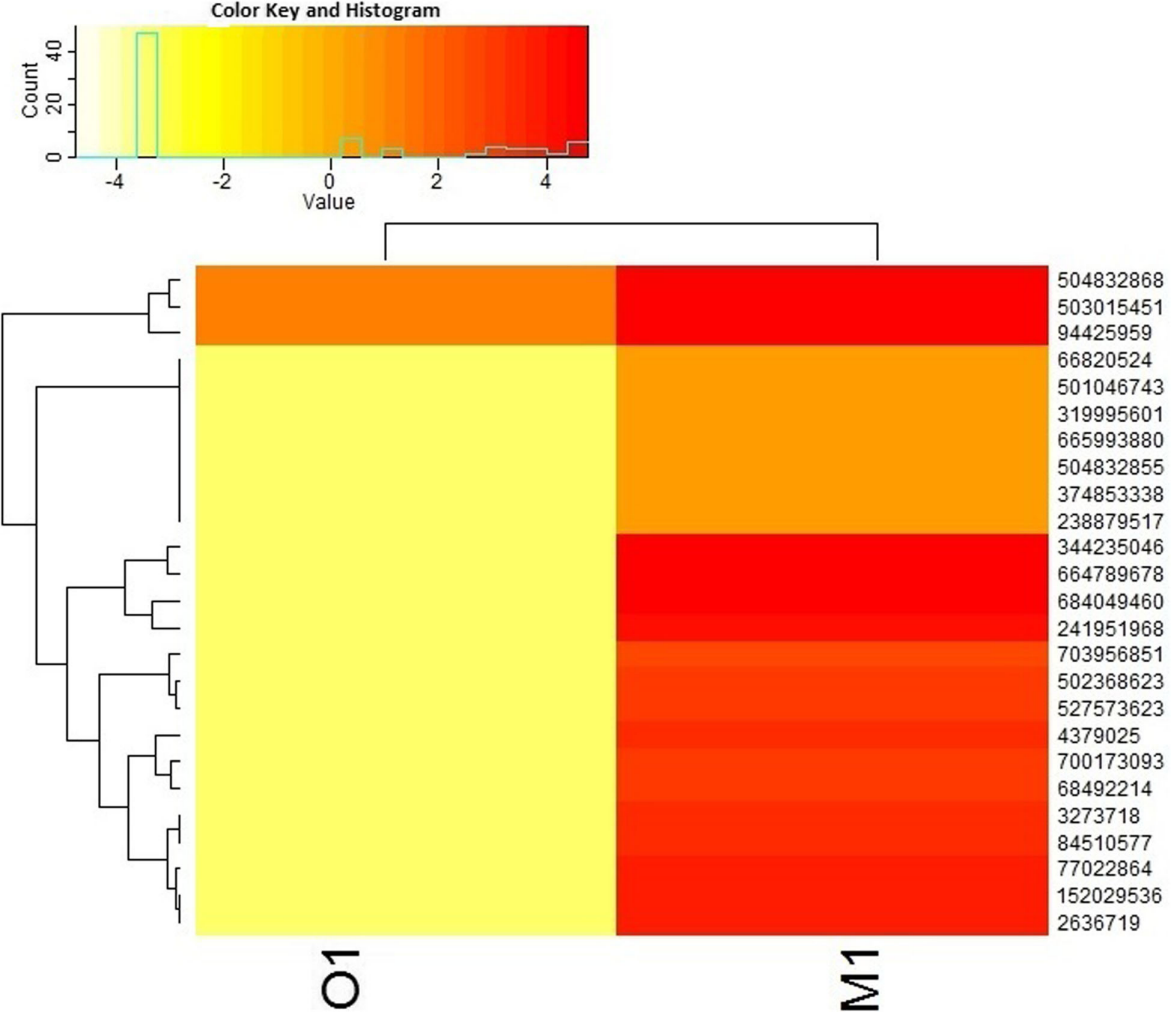
Heat map showing expression of top 25 expressed genes in M1 compared to O1, with *p* value ≤ 0.05. The details description of the gene IDs is shown in Table 2

High-quality RNA was extracted from agricultural soil (M1) and organic soil (O1) and was quantified using Nanodrop. Paired-end Illumina cDNA libraries from two datasets yielded ~ 11.49 GBp and ~ 11.9 GBp data for the M1 and O1 samples, respectively (Table 1). A total of 114,908,828 and 119,019,498 raw data (R1 and R2) was sequenced for both M1 and O1respectively. The sequencing data showed 55% GC content and a Phred score ≥ 30. After removal of adapter and non-coding RNA, we assembled the metatranscriptome sequencing reads; however, very less number of reads could be assembled due to complexity and heterogeneity in the metatranscriptome datasets (data not shown). Alternatively, we used a read-based annotation after sequence clustering, as recommended previously [27, 28]. Clustering of sequences resulted in ~ 32,768 representative mRNA reads for M1 and 5611 for O1 respectively. The average length of the mRNA representative reads was > 100 and < 250 bp. Read-based annotation of clustered sequences were classified according to cellular (GO 00005575), molecular (GO 0003674), and biological (GO 0008150) processes [28]. Altogether, from GO assignments, it is evident that the two soil types share similar molecular function; however, they displayed a significant level of variations with respect to cellular and biological function. Percent distribution of transcripts assigned to biological, cellular, and molecular functions among the two datasets is shown in Fig. 1. Gene Ontology (GO) assignments and taxonomical classification are depicted in Additional file 3: Table S1 and Additional file 4: Table S2. The two soil systems however demonstrated great variations for processes involving energy generation, metabolism, transportation, and ribosomal activity. High ATPase and GTPase activity is directly related to energy, motility, host-pathogen interactions, and export of toxins and other wastes out of the cell and in multidrug resistance [29] The iron and sulfur binding metalloproteases play important roles in cellular and biological activities in both prokaryotes and eukaryotes [30]. Several transcripts playing crucial role in oxidation-reduction processes showed enhanced expression in M1. The top 25 transcripts from various organisms demonstrating abundant expression in M1 compared to O1 are depicted in Fig. 2 and Table 2. Other differentially expressed genes enlisted in Additional file 5: Table S3 were also analyzed to understand various metabolic processes taking place in soil.

**Table 1:** Description of the raw reads, percent of GC content, and quality score

Sample ID	Total reads (R1 and R2)	Sequence length (bp)	Total data (Gb)	Percent of GC content	Percent of data ≥ 30 Phred score
M1	114,908,828	100 × 2	~ 11.49	55	≥ 30
O1	119,019,498	100 × 2	~ 11.9	55	≥ 30

**Table 2:** Detailed description of the top 25 high expressed genes in M1 compared to O1 (*p* value ≤ 0.05)

Transcript_ID	UNIPROT ID	Protein names	Gene names	Organism	Gene length
504832868	K0IHK8	Monooxygenase, subunit *C*	amoC1 Ngar_c25120	*Nitrososphaera gargensis* (strain Ga9.2)	184
503015451	D8P7X5	Uncharacterized protein	NIDE3937	*Candidatus Nitrospira defluvii*	1645
94425959	Q1MYB0	Uncharacterized protein	RED65_03040	*Bermanella marisrubri*	119
66820524	Q553U6	Putative actin-22	act22 DDB_G0275023	*Dictyostelium discoideum* (slime mold)	376
501046743	A7HGP7	10 kDa chaperonin (GroES protein)	groS groES Anae109_3714	*Anaeromyxobacter* sp. (strain Fw109-5)	97
319995601	E8MYN6	Cell division protein FtsZ	ftsZ ANT_23460	*Anaerolinea thermophila*	387
665993880	A0A075MRB7	Cytochrome *b* subunit of the bc complex	NTE_01577	*Candidatus Nitrososphaera evergladensis SR1*	546
504832855	K0ILC3	Methylmalonyl-CoA mutase, small subunit	Ngar_c24990	*Nitrososphaera gargensis* (strain Ga9.2)	141
374853338	H5SJB3	MFS transporter	HGMM_F35G12C20 HGMM_F51D07C02	Uncultured *Acetothermia bacterium*	412
238879517	C4YNC2	Phosphoenolpyruvate carboxykinase	CAWG_01381	*Candida albicans* (strain WO-1) (yeast)	553
344235046	G3IQD4	Putative uncharacterized protein	I79_026257	*Cricetulus griseus* (Chinese hamster) (*Cricetulus barabensis griseus*)	60
664789678	A0A075KDX2	Uncharacterized protein	UFO1_2808	*Pelosinus* sp. *UFO1*	128
684049460	A0A094MWR7	Uncharacterized protein	Thpro_01067	*Thiobacillus prosperus*	71
241951968	B9WC89	d-arabinose dehydrogenase	CD36_22310	*Candida dubliniensis* (strain CD36/ATCC MYA*-*)	326
703956851	A0A0A3BUY2	Uncharacterized protein	MEO_05149	*Candida albicans P94015*	1541
502368623	C6E7X5	Multicopper oxidase type 2	GM21_0071	*Geobacter* sp. (strain M21)	1299
527573623	S8JMM6	Endonuclease	SAG0065_03165	*Streptococcus agalactiae CCUG 37742*	1275
4379025	O95662	KpnI repetitive sequence		*Homo sapiens* (human)	194
700173093	A0A0A0JA99	Uncharacterized protein	N798_05235	*Knoellia flava TL1*	1571
68492214	Q59K39	Retrotransposon Tca2	POL98	*Candida albicans*	1566
3273718	O74209	Pol polyprotein (fragment)	pol	*Candida albicans* (yeast)	1576
84510577	A3V3H5	Uncharacterized protein	SKA53_01511	*Loktanella vestfoldensis SKA53*	91
77022864	Q3MP87	Putative uncharacterized protein CaJ7.0300	CaJ7.0300 CaO19.6451	*Candida albicans* (yeast)	1566
152029536	A7HF08	Multicopper oxidase type 2	Anae109_3108	*Anaeromyxobacter* sp. (strain Fw109-5)	1430
2636719	O13308	POL protein (fragment)		*Candida albicans* (yeast)	1576

### Phylogeny analysis

**Fig. 3:**
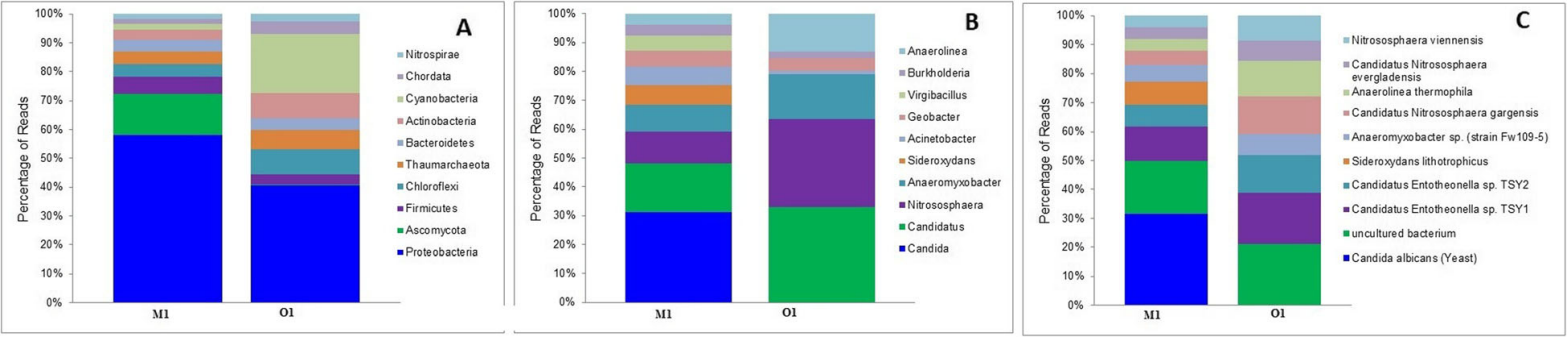
Percent distribution of transcripts representing various taxonomical groups at phylum, genus, and species level in M1 and O1. **a** Phylum. **b** Genus. **c** Species

Taxonomic analysis (Additional file 3: Table S1) of the annotated reads, as presented in Fig. 3, revealed *Proteobactreria* as major phylum in the two soil *types,* the order of top 3 abundant phyla in the M1 was *Proteobacteria* > *Ascomycota* > *Firmicutes*, whereas sample O1 demonstrated the following order *Proteobacteria*>*Cyanobacteria*>*Actinobacteria* (Fig. 3a). *Proteobacteria* encompass enormous diversity in terms of physiology, morphology, and metabolism and is considered as a key player in carbon and nitrogen cycle. *Deltaproteobacteria* and *Alphaproteobacteria* showed abundant presence in M1, whereas *Lilliopsida* and *Deltaproteobacteria* were more in O1. Another important phylum identified in this M1 soil sample belongs to *Thaumarchaeota*. At the genus level, ~ 1312 transcripts were assigned to the M1 sample, and the most dominant genus identified was *Candida* (30%) followed by *Candidatus* and *Nitrosophaera* in M1 (Fig. 3b). At the species level, *Candida albicans* (30%) and *Sideroxydans lithotrophicus* (8%) revealed their unique occurrence in M1. Interestingly, *Anaeromyxobacter* sp. Fw109-5, *Candidatus Nitrososphaera gargensis*, *N. Viennensis*, *Anaerolinea thermophila*, *Candidatus Entotheonella* sp. *TSY1*, and *Candidatus Entotheonella* sp. *TSY2* were less represented in the M1 sample as compared to O1 (Fig. 3c)*.*

### Comparing nitrification process in agriculture (M1) and organic soil (O1)

Nitrification is used to monitor the side effects of contaminants on microbial community structure, function, and soil quality. KEGG (Kyoto Encyclopedia of Genes and Genomes analysis) analysis of nitrification pathway revealed that several transcripts encoding genes of nitrification were highly expressed in M1 that mainly include *amoC* from AOA (ammonia-oxidizing archaea) (*amoC1*, *C2* = 4.3, 4.17-fold in *N. gargensis*, *amoC3* = 1.46 in *Nitrososphaera viennensis EN76*, *NTE_00725* = 3, *NTE_01100* = 1.44-fold in *Candidatus Nitrososphaera evergladensis SR1*), indicating active contribution of AOA in nitrification process in M1 soil. Furthermore, we observed a high expression of putative *NxrA2* and *NxrB1/B2*, i.e., nitrite oxidoreductase (*NXR*), in the M1 soil sample (Additional file 4: Table S4) from *C. Nitrospira defluvii* and a beta subunit of nitrite oxidoreductase from *Nitrospira moscoviensis*, a key enzyme of NO_2_ oxidation in NOB (nitrate-oxidizing bacteria). In addition, copper-containing nitrite reductases (*NirK*) from uncultured bacterium also had high expression in the M1 soil. Interestingly, a membrane-bound dissimilatory nitrate reductase (~ 2.7-fold) *Tbd_1403* of *T. denitrificans* was also highly expressed in agriculture soil M1. High expression of the small subunit of methylmalonyl-CoA mutase (EC 5.4.99.2) that convert malonyl-CoA to succinyl-CoA as the main product of an alternative carbon fixation pathway was also observed in M1.

### Analysis of stress-related transcripts

**Fig. 4:**
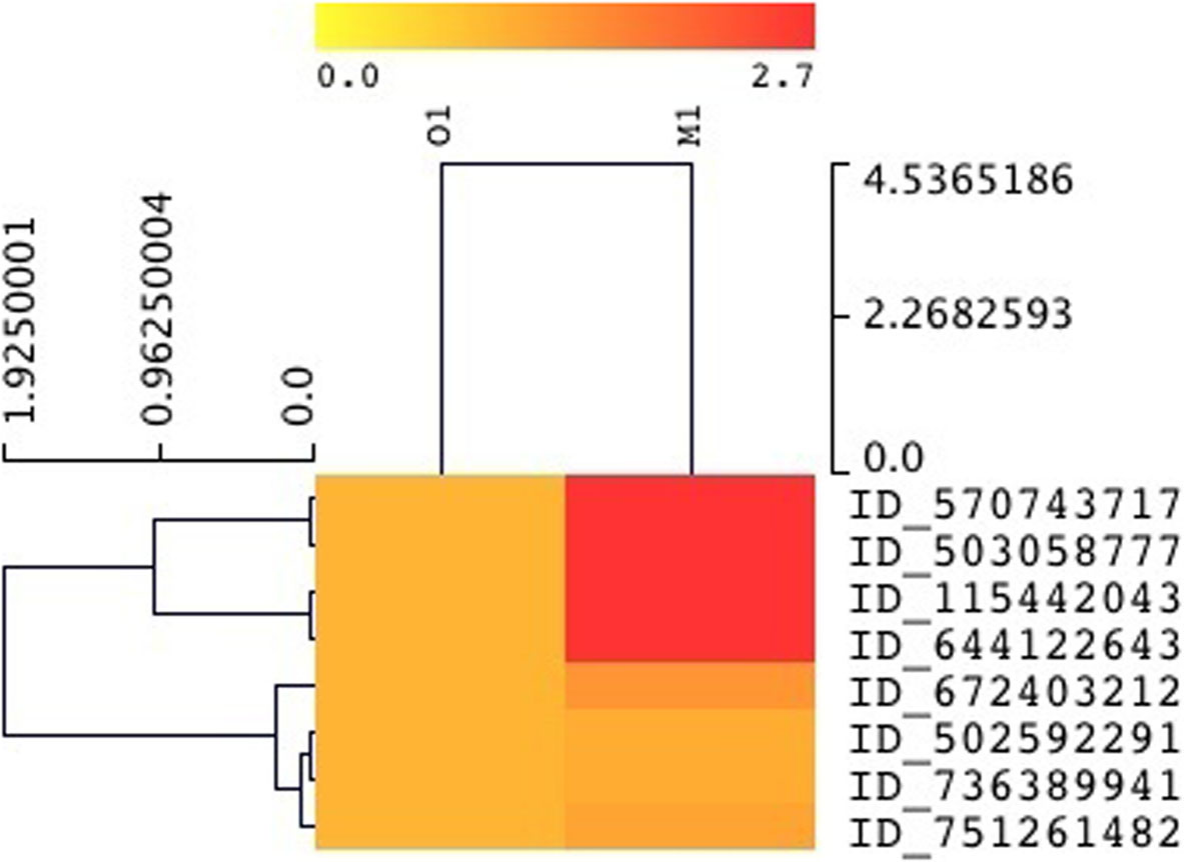
Heat map showing high expression of heat shock proteins in the M1 soil, refer to Additional file 4: Table S2 for details

Several chaperones demonstrated high expression (~ 1.6–3-fold increase, Fig. 4 and Additional file 3: Table S2) that include DnaK (HSP70) (heat shock 70 kDa protein) with transcript ID 570743717, 10 kDa chaperonin (GroES protein) transcript ID 644122643 (Protein Cn10), ATP-dependent chaperone ClpB transcript ID 503058777, 60 kDa chaperonin (GroEL protein) transcript ID 751261482, and ATPase with chaperone activity and a copper chaperones (CCH-related protein) with transcript ID_11542043. Additionally, several proteases also showed high expression in the polluted soil sample that includes Lon protease (EC 3.4.21.53) (ATP-dependent protease La) of *Candidatus Entotheonella* sp., serine protease of *Nitrosococcus oceani*, ATP-dependent zinc metalloprotease FtsH (EC 3.4.24.-) ftsH from *Nitrosospira*, ATP-dependent zinc metalloprotease FtsH (EC 3.4.24.-) ftsH from *Caldilinea aerophila*, carboxyl-terminal protease (EC 3.4.21.102) of *Niastella koreensis*, ATP-dependent zinc metalloprotease FtsH (EC 3.4.24.-) ftsH of *Anaerolinea thermophila*, and Lon protease (EC 3.4.21.53) (ATP-dependent protease La) of *Anaeromyxobacter* sp. Abundant expression of transcripts encoding endonuclease (transcript ID_527573623) from *Streptococcus agalactiae* indicates heavy metal contamination in the M1 soil sample, in which both Ni and Cd potentially affect the activity of LINE-1.

### Aromatic and hydrocarbon metabolism

**Fig. 5:**
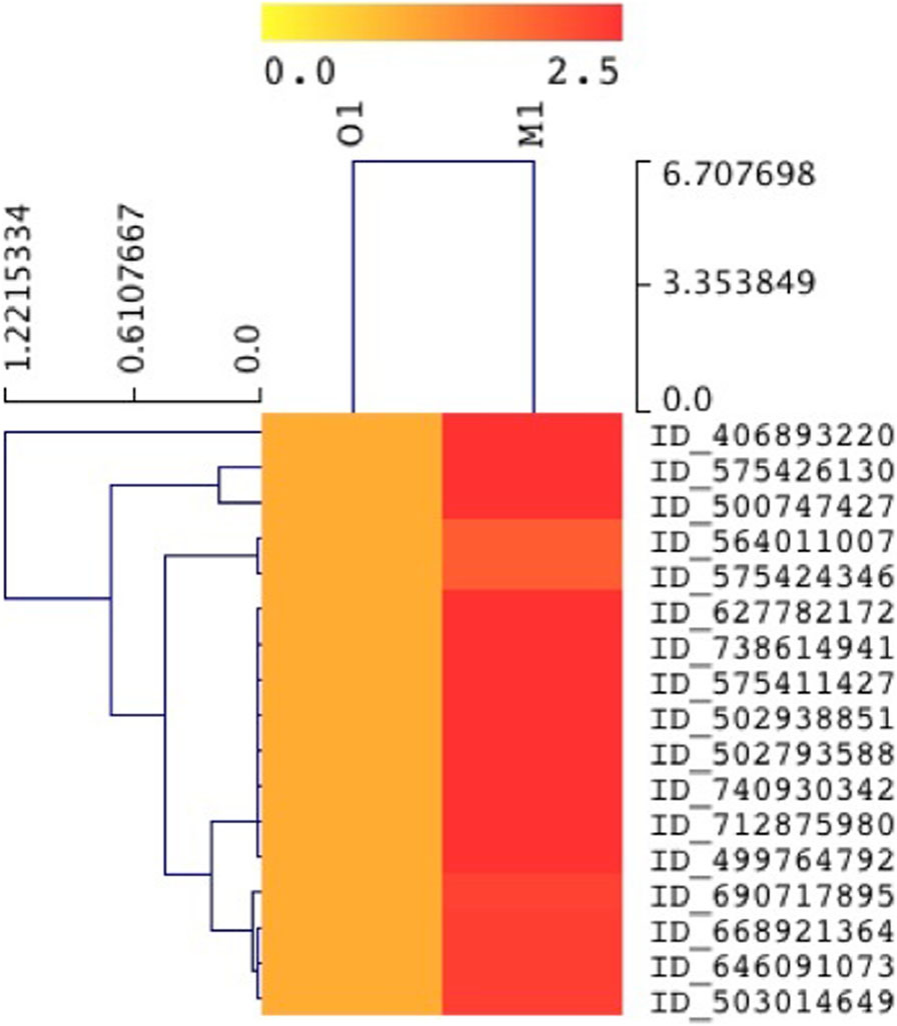
Heat map showing expression of genes involved in aromatic compound degradation in M1 compared to O1 with *p* value ≤ 0.05. The detailed description of the genes is provided in Table 3

Transcripts associated with the metabolism of aromatic hydrocarbon demonstrated their high expression in cypermethrin- and metal-contaminated M1 soil (Fig. 5 and Table 3) compared to O1. Increased expression of transcripts related to aromatic metabolism was widespread and associated with both central and peripheral aromatic metabolic pathways. Interestingly, high expression of transcripts that include 4-hydroxyphenylpyruvate dioxygenase (HPPD), glyoxalase/bleomycin resistance protein/dioxygenase, metapyrocatechase, and some uncharacterized protein share similarity with vicinal oxygen chelate superfamily (VOC family). Another transcript-encoding 2-nitropropane dioxygenase [EC 1.13.12.16] also showed high expression in M1 sample; this enzyme catalyzes oxidation of nitroalkanes into their corresponding carbonyl product. Nitronate monooxygenase (NMO), formerly referred as 2-nitropropane dioxygenase (NPD) (EC 1.13.11.32), is an FMN-dependent enzyme that uses molecular oxygen to oxidize (anionic) alkyl nitronates. Of the particular interests, we observed high expression of transcripts encoding ring hydroxylating dioxygenases (EC 1.14.12.12) from *Novosphingobium aromaticivorans* (*strain DSM 12444*/*F199*) and intradiol ring-cleavage dioxygenase from *Novosphingobium resinovorum*.

**Table 3:** Transcripts showing high expression related to aromatic hydrocarbon degradation in M1 compared to O1 (*p* value ≤ 0.05)

Transcript_ID	UniProt ID	Protein names	Gene names	Organism	Gene length
627782172	A0A023XH36	Metapyrocatechase	BJS_04241	*Bradyrhizobium japonicum SEMIA 5079*	326
668921364	A0A085DUZ9	Protocatechuate 3,4-dioxygenase	DK37_06335	*Halomonas* sp. *SUBG004*	196
738614941	A0A031K144	Ring hydroxylating dioxygenase, alpha subunit	BV97_01833	*Novosphingobium resinovorum*	425
575411427	W4LH05	Uncharacterized protein	ETSY1_22950	*Candidatus Entotheonella* sp. *TSY1*	171
575426130	W4MGP2	Uncharacterized protein	ETSY2_00190	*Candidatus Entotheonella* sp. *TSY2*	169
564011007	W9BZ28	2-Nitropropane dioxygenase	Q27BB25_02350	*Blastomonas* sp. *CACIA14H2*	467
575424346	W4MC15	Uncharacterized protein	ETSY2_08610	*Candidatus Entotheonella* sp. *TSY2*	380
502938851	D6XZD0	2-Nitropropane dioxygenase NPD	Bsel_2926	*Bacillus selenitireducens*	318
502793588	D5CM51	2-Nitropropane dioxygenase NPD	Slit_0424	*Sideroxydans lithotrophicus* (strain ES-1)	416
646091073	A0A069IMT4	4-Hydroxyphenylpyruvate dioxygenase	ER13_02875	*Brevundimonas* sp. *EAKA*	373
500747427	A7HAK0	Glyoxalase/bleomycin resistance protein/dioxygenase	Anae109_1540	*Anaeromyxobacter* sp. (strain Fw109-5)	121
740930342	A0A063B6R9	Glyoxalase/bleomycin resistance protein/dioxygenase	LIG30_4306	*Burkholderia* sp. *lig30*	183
406893220	K1Z0Y1	Glyoxalase/bleomycin resistance protein/dioxygenase	ACD_75C00771G0006	Uncultured bacterium	157
690717895	A0A098SBR0	Homogentisate 1,2-dioxygenase	IX84_02245	*Phaeodactylibacter xiamenensis*	390
712875980	A0A0A4BJC1	Indoleamine 2,3-dioxygenase	MG7_04021	*Candida albicans P34048*	440
499764792	Q2G757	Intradiol ring-cleavage dioxygenase	Saro_1876	*Novosphingobium aromaticivorans* (strain DSM 12444/F199)	313
503014649	D8PHR7	Putative homogentisate 1,2-dioxygenase (EC 1.13.11.5)	NIDE3110	*Candidatus Nitrospira defluvii*	371

## Discussion

Investigation of transcripts encoding functional protein by direct sequencing and analysis at a particular time and space can disclose structural and functional features of microbial communities. In this study, analysis of metatranscriptome samples from two distinct soils revealed divergences in functional features of microbial communities. Interestingly, during phylogeny analysis, we observed an abundant presence of archaea especially *Thaumarchaeota*, a phylum which is widely distributed in extreme, non-extreme [31], terrestrial [32], and metal-contaminated soil [33]; these observations primarily indicate important consequences of archaea, especially towards degradation of the pesticides, ammonia oxidation [34], nitrate leaching in greenhouse gas production, and soil subsidence [35]. Additionally, the abundance of *Candida albicans* and *Sideroxydans lithotrophicus* in the M1 soil sample suggests their high tolerance to metals as also reported previously [36]. It was interesting to note that despite of low abundance of archaea in the M1 soil, it has shown high expression of genes related to several pathways, thus indicating archaeal and better adaptation and active contribution in pesticide- and metal-contaminated environment, e.g., several transcripts related to nitrification process, which is used to monitor side effects of pesticide contamination [37], showed high expression in M1. A highly expressed *amoC* transcript identified in the M1 soil (belongs to group I.1.a of *archaea* as depicted in Additional file 1: Figure S1, which harbor an additional copy of *amoC* gene) is well correlated previously with high metal resistance [38–40]. Additionally, we observed high expression of putative *NxrA2* and *NxrB1/B2*, i.e., nitrite oxidoreductase (*NXR*) (Additional file 4: Table S4), from *C. Nitrospira defluvii* and *β* subunit of nitrite oxidoreductase from *Nitrospira moscoviensis*, key enzymes of NO_2_ oxidation in NOB indicating active participation of these organisms in nitrification in M1 soil. On further analysis, we found that these genes have shared similarity with *NxrA2* and *NxrB* of *N. moscoviensis* and with other known NO_2_^−^/NO_3_^−^-binding molybdoenzymes, such as *NXR* of *Nitrobacter* or bacterial nitrate reductases (*NARs*) which shuttles two electrons per oxidized NO_2_ into the electron transport chain [41]. Another transcript (*NirK*) from uncultured bacterium also had high expression in M1; this transcript has shared maximum homology (> 70%) with *NirK* of *Chloroflexi* thus indicating denitrification through AOB (ammonia-oxidizing bacteria) [42] that forms NO from NO_2_ [43]. Besides *nirK* homologs, no other genes typically contributing to the denitrification process showed high expression in the M1 soil. We therefore propose that high expression of transcripts *nirK* along with *amoC* can be a useful molecular marker to monitor soil ammonia oxidation in agricultural soil contaminated with pesticides and metals. Additionally, high expression (~ 2.7-fold) of a membrane-bound dissimilatory nitrate reductase *Tbd_1403* of *T. denitrificans* in agriculture soil indicates nitrate-dependent oxidation of metals with high reduction potentials and can also be used as molecular marker to study metal resistance pathways among denitrifying bacteria showing high metal tolerance U(IV) and Fe(II) [44–46].

In addition to high expression of transcripts related with nitrification and ammonia oxidation from *Thaumarchaeal* viz *N. gargensis*, we observed high expression of PCK1 in archaea; PCK1 regulates 3-hydroxypropionate/4-hydroxybutyrate carbon fixation pathway as reported previously [47–50]. In particular, high expression of PCK1 gene from *C. albicans* that encodes protein of gluconeogenesis pathway indicate soil environment where carbon availability might be changing continuously enabling *C. albicans* to switch its metabolism by regulating PCK1, as also reported previously in metal-contaminated soil [51, 52]. Another transcript that demonstrated high expression in M1 belongs to a putative NADP^+^-specific d-arabinose dehydrogenase from *C. dubliniensis* sharing > 95% identity with *C. albicans*. The exact mechanism of action of d-arabinose dehydrogenase is unknown; however, its activity is shown to be inhibited by metals presence including Hg^2+^ [53]; thus, it may be hypothesized that high expression of d-arabinose dehydrogenase is indicating oxidative stress response of *C. albicans* in agriculture soil*.*

Microbes demonstrate multiple adaptations to survive under osmotic, oxidative, heavy metals, elevated temperatures, high salinity, and other stress conditions, one of the strategies that microbes employ to cope up these stresses is accumulation of compatible solutes and small soluble organic molecules [54]. In order to investigate the transcripts showing high expression in metal and pesticide stress conditions, we compared and analyze the transcripts associated with stress from two soil types; interestingly, high expression of UDP-glucose 6-dehydrogenase (EC 1.1.1.22) (udg_NIDE4145; ~ 2.7-fold) and pyruvate to ferredoxin oxidoreductase *δ* subunit (EC 1.2.7.1) (porD_NIDE0972; ~ 1.7-fold) indicate active synthesis of glycogen in Ca. *N. defluvii* in response to stress [38]. These solutes can either be transported into the cell or synthesized de novo. Indeed, glycogen deposits have been observed under electron microscopy in *Nitrospira* cells under stress conditions [55]. To mitigate oxidative damage, in contrast to most of the aerobic bacteria, *N. defluvii* lacks SOD and catalase, and its genome lacks superoxide dismutase (SOD) either. High expression of bacterioferritin transcripts in Ca. *N. defluvii* indicates ROS (reactive oxygen species) detoxification in M1 soil; it binds with free iron thus reduces the risk of ROS generation. Additionally, transcript ID_664789678 (Table 2) with unknown function from *Pelosinus* species also showed high expression in the M1 soil sample compared to O1. Previously, this organism has shown its presence in various sites that include Melton Branch Watershed, Oak Ridge, TN, USA ([56] and uranium-contaminated field site near Oak Ridge [57]. During its growth, it uses Fe(III) [58] and U(VI) [59]. As most of the proteins from *Pelosinus* are not characterized [60], we hypothesized that high expression of one of its transcripts in the M1 soil can be correlated with the presence of heavy metals in M1 compared to the O1 soil. Additionally, high expression of cell division-related protein *FtsZ* from *Anaerolinea thermophila* indicates active peptidoglycan biosynthesis in the M1 soil. Furthermore, a multicopper oxidase (transcript ID_ 502368623, 700173093, 152029536) from *Geobacter* sp., *Knoellia* sp., and *Anaeromyxobacter* sp. also showed high expression in the M1 soil. Catalytic sites of these proteins contain copper-binding sites and are implicated in bacterial copper resistance, oxidation of phenolic compounds, and in detoxification of Mn^2+^ [61]. Additionally, high expression of putative actin 22 in M1 indicates the formation of actin rods which was previously reported in *Dictyostelium* during the formation of spores under stress conditions such as heat shock and osmotic pressure as well as non-nutrient conditions [62, 63]. Many of these chaperones were of *Proteobacterial* origin and of high expression. These proteases were also of proteobacterial origin, and their role has not yet established in the metal- and pesticide-contaminated agriculture soil; however, we propose that their high expression can be correlated with high turnover in *Proteobacteria* which mostly depends on ATP-dependent proteases that are recruited by the cells in the cytosol (Lon, ClpAP, ClpXP, HslVU) and are well reported in cypermethrin- and cadmium-contaminated soil [64–67] or are associated with the inner membrane (FtsH) [68]. These enzymes in adverse conditions not only help in degradation of misfolded or abnormal proteins but also degrade unstable proteins, like σ^32^ heat shock factor. Another transcript *Tca2* which is widely distributed in *C. albicans*, related with Ty1/copia-type retrotransposon [69], and is predominantly present in the M1 soil (transcript ID_68492214, 77022864, 703956851, 3273718) support the fact that *Tca2* transpositional activity is favored at high-temperature stress and reveal a close relationship between the *Tca2* expression and virulence in *C. albicans* endonuclease activity in vitro since it also utilizes Mg^2+^ as a cofactor for enzymatic activity [70]. It further raises the possibility of co-selection of antibiotic and heavy metal resistance dissemination through mobile genetic elements [71] observed in other transcripts in the current study. High expression of MFS (transcript id_374853338) in uncultured *Acetothermia* bacterium showing similarity with oxalate/formate antiporter of proteobacteria, i.e., *Nitrosomonas* sp., point out that it may be participating in exchange of external divalent oxalate with the intracellular monovalent formate [72, 73] to generate a proton-motive force that supports membrane functions, including ATP synthesis, accumulation of growth substrates, and extrusion of waste products [72–75].

Furthermore, the analysis of metabolic pathways associated with aromatic hydrocarbon metabolism revealed high expression of glyoxalase/bleomycin resistance protein/dioxygenase, metapyrocatechase, and some uncharacterized protein sharing similarity with vicinal oxygen chelate superfamily (VOC family) that is known to harbor a β-α-β motif in cypermethrin- and metal-contaminated soil (Fig. 5 and Table 3); similar observations were also reported in previous studies [76, 77]. This β-α-β motif provides a metal coordination environment for these enzymes and helps them in catalyzing different enzymatic reactions including isomerizations in glyoxalase I and epimerization in methylmalonyl-CoA epimerase, in oxidative cleavage of C-C bonds in extradiol dioxygenase and nucleophilic substitutions in fosfomycin/bleymucin resistance proteins [78]. Phylogenetically, these transcripts showed their origin from *Geobacter*, strains viz. *G. metallireducens*, *G. pickeringii*, and *Geobacter uraniireducens* (Additional file 2: Figure S2), which are often the dominant members of subsurface sediments, dwell under metal-reducing conditions [79], retrieve energy through dissimilatory reduction of metals ions, and efficiently oxidize monoaromatic compounds such as toluene and phenol [80, 81]. We have also observed high expression of transcripts encoding enzyme 2-nitropropane dioxygenase [EC 1.13.12.16], an enzyme catalyzing oxidation of nitroalkanes into their corresponding carbonyl product. Nitronate monooxygenase (NMO), formerly referred as 2-nitropropane dioxygenase (NPD) (EC 1.13.11.32) which oxidizes (anionic) alkyl nitronates, upregulation of NPD gene in the agriculture soil M1, indicates high nitronate contamination in cypermethrin- and metal-contaminated soil. Similar observations were reported previously where authors have shown upregulation of NPD in *Sideroxydans lithotrophicus* (*strain ES*-*1*) in iron-contaminated groundwater [82] indicating its stimulatory effect with Fe^2+^ in the agricultural soil [83–85]. Of the particular interests, high expression of transcripts encoding ring hydroxylating dioxygenases (EC 1.14.12.12) from *Novosphingobium aromaticivorans* (*strain DSM 12444*/*F199*) and intradiol ring-cleavage dioxygenase from *Novosphingobium resinovorum* indicate active participation of these organisms in the degradation process of polycyclic aromatic hydrocarbon that includes naphthalene and ethylbenzene [86–90].

## Conclusion

In summary, a comparative metatranscriptomic study from agriculture and organic soil types illuminated structural and functional variations. The study further concluded that high expression of microbial transcripts in two soil types is associated with wide range of functions. It has also provided clue about several molecular markers which could be a strong indicator of metal and pesticide contamination in soils. Interestingly, our study revealed that archaea are playing a significant role in nitrification process compared to bacteria in metal- and pesticide-contaminated soil. In particular, high expression of transcripts related to aromatic hydrocarbon degradation provided clue about degradation potential associated in polluted soil communities.

## Supplementary information

**Additional file 1: Fig. S1**. Phylogenetic analysis of upregulated *amoC* genes obtained using neighbor-joining clustering method having bootstrap values out of 1000 replicates using MEGA 7.0 using UniProt ID with their species and gene name (GN). K0IHK8_ *Nitrososphaera gargensis* (strain Ga9.2)_amoC1, A0A075MNL6_ *Candidatus Nitrososphaera evergladensis* SR1_NTE00725, A0A060HQE0_ *Nitrososphaera viennensis* EN76_amoC3, A0A075MPN8_ *Candidatus Nitrososphaera evergladensis* SR1_ NTE_01100. It clearly indicates that thaumarchaea species harbor additional copy of *amoC* that could be used as a molecular marker in the detection of particular archeal community present in cypermerthrin and metal co-contaminated agriculture soils i.e. M1 soil sampl**e. (JPG 82 kb)**

**Additional file 2: Fig. S2.** Phylogenetic analysis of VOC superfamily transcript obtained in M1 soil sample using neighbor-joining clustering method having bootstrap values out of 1000 replicates using MEGA 7.0 using UniProt ID with their species (K1Z0Y1_uncultured bacterium, A0A063B6R9_ *Burkholderia* sp. lig30,A7HAK0_ *Anaeromyxobacter* sp. (strain Fw109-5), A0A069IMT4_ *Brevundimonas* sp. EAKA, A0A023XH36_ *Bradyrhizobium japonicum* SEMIA 5079) that clearly indicates the evolution of VOC superfamily mostely from *Geobacter* sp., *Nitrospirae* sp. *Deulphobulbacae sp, actinobacteria* sp.*, candidatus Entothella* sp. present dominantly in M soil sample. (JPG 122 kb)

**Additional file 3: Table S1**. The transcript id, gene function, gene ontology (biological,molecular and cellular function) obtained M1 functional hits after quarried against UniPrt database. (XLS 5019 kb)

**Additional file 4: Table S2**. The transcript id, gene function, gene ontology (biological, molecular and cellular function) obtained O1 functional hits after quarried against UniProt database. (XLS 1457 kb)

**Additional file 5: Table S3.** List of Differentially Expressed genes in M1 and O1sample. (DOC 45 kb)

## Data Availability

The metatranscriptome data for M1 and O1 is submitted with MG-RAST under the accession number mgm4653349.3, whereas M1 dataset was provided a Gold ID 0eeec568676d676d343733323034392e33.
