# Supplementary figures and images for: Comparative metatranscriptome analysis revealed broad response of microbial communities in two soil types, agriculture versus organic soil
Source: J Genet Eng Biotechnol. 2019 Oct 14;17:6. doi: 10.1186/s43141-019-0006-3 (PMC6821142; doi:10.1186/s43141-019-0006-3)

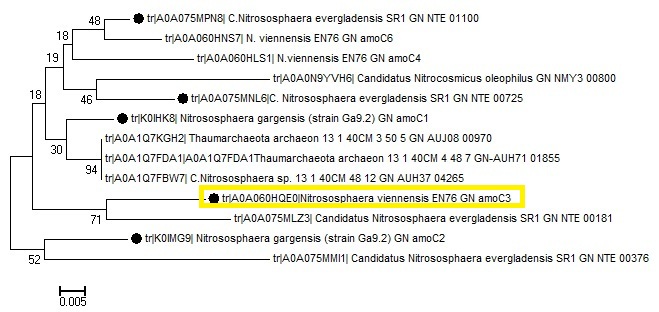

Supplement: Supplementary file 1 — Additional file 1: Fig. S1. Phylogenetic analysis of upregulated amoC genes obtained using neighbor-joining clustering method having bootstrap values out of 1000 replicates using MEGA 7.0 using UniProt ID with their species and gene name (GN). K0IHK8_ Nitrososphaera gargensis (strain Ga9.2)_amoC1, A0A075MNL6_ Candidatus Nitrososphaera evergladensis SR1_NTE00725, A0A060HQE0_ Nitrososphaera viennensis EN76_amoC3, A0A075MPN8_ Candidatus Nitrososphaera evergladensis SR1_ NTE_01100. It clearly indicates that thaumarchaea species harbor additional copy of amoC that could be used as a molecular marker in the detection of particular archeal community present in cypermerthrin and metal co-contaminated agriculture soils i.e. M1 soil sample. (JPG 82 kb) [file 43141_2019_6_MOESM1_ESM.jpg]

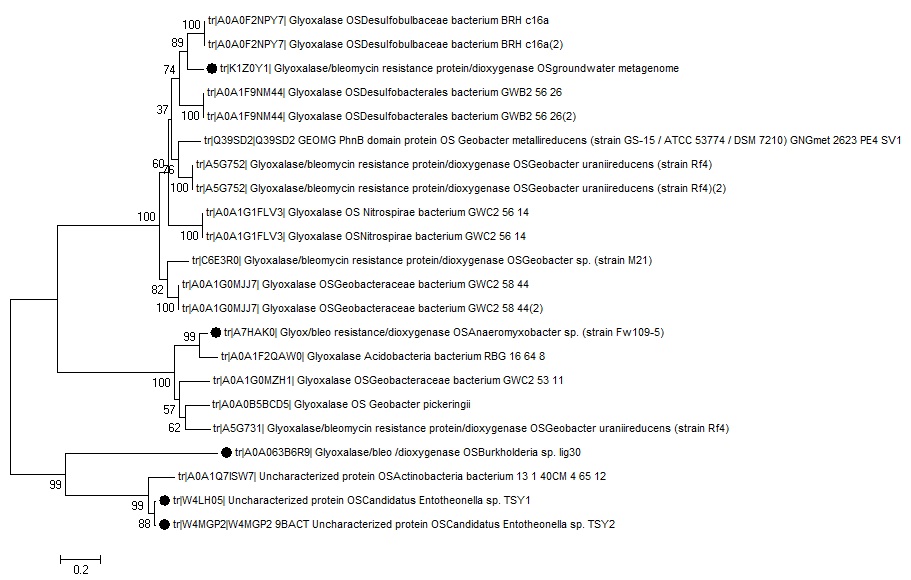

Supplement: Supplementary file 2 — Additional file 2: Fig. S2. Phylogenetic analysis of VOC superfamily transcript obtained in M1 soil sample using neighbor-joining clustering method having bootstrap values out of 1000 replicates using MEGA 7.0 using UniProt ID with their species (K1Z0Y1_uncultured bacterium, A0A063B6R9_ Burkholderia sp. lig30,A7HAK0_ Anaeromyxobacter sp. (strain Fw109-5), A0A069IMT4_ Brevundimonas sp. EAKA, A0A023XH36_ Bradyrhizobium japonicum SEMIA 5079) that clearly indicates the evolution of VOC superfamily mostely from Geobacter sp., Nitrospirae sp. Deulphobulbacae sp, actinobacteria sp., candidatus Entothella sp. present dominantly in M soil sample. (JPG 122 kb) [file 43141_2019_6_MOESM2_ESM.jpg]
