# Supplementary material for: Comparative metatranscriptome analysis revealed broad response of microbial communities in two soil types, agriculture versus organic soil
Source: J Genet Eng Biotechnol. 2019 Oct 14;17:6. doi: 10.1186/s43141-019-0006-3 (PMC6821142; doi:10.1186/s43141-019-0006-3)
Supplement: Supplementary file 5 — Additional file 5: Table S3. List of Differentially Expressed genes in M1 and O1sample. (DOC 45 kb) [file 43141_2019_6_MOESM5_ESM.doc]

| **Supplementary Table S3.** Differentially Expressed genes | | | | | | | | |
| --- | --- | --- | --- | --- | --- | --- | --- | --- |
| Sr.No. | **Transcript_ID** | **foldChange (O1/M1) P value ≤ 0.05** | **log2FoldChange (O1/M1) P value ≤ 0.05** | **UniProt_ID** | **Protein names** | **Gene names** | **Organism** | **Gene Length** |
|  |  |  |  |  |  |  |  |  |
| 1 | 665993043 | 0.125 | -3 | A0A075MNL6 | Ammonia monooxygenase/methane monooxygenase, subunit C | NTE_00725 | *Candidatus Nitrososphaera evergladensis SR1* | 184 |
| 2 | 647811686 | 0.361111111 | -1.469485283 | A0A060HQE0 | Ammonia monooxygenase subunit C | amoC3 NVIE_011560 | *Nitrososphaera viennensis EN76* | 187 |
| 3 | 665993413 | 0.367698901 | -1.443403228 | A0A075MPN8 | Ammonia monooxygenase/methane monooxygenase, subunit C | NTE_01100 | *Candidatus Nitrososphaera evergladensis SR1* | 187 |
| 4 | 503014791 | 0.590944663 | -0.758905054 | D8PI59 | Putative Nitrate oxidoreductase, alpha subunit (EC 1.7.99.4) | nxrA2 NIDE3255 | *Candidatus Nitrospira defluvii* | 1147 |
| 5 | 503014772 | 0.67405237 | -0.569067409 | D8PI40 | Putative Nitrate oxidoreductase, beta subunit (EC 1.7.99.4) | nxrB1 nxrB2 NIDE3236 NIDE3256 | *Candidatus Nitrospira defluvii* | 429 |
| 6 | 374855677 | 0.928571429 | -0.106915204 | H5SR15 | Nitrous-oxide reductase | HGMM_OP2C082 | *Candidatus Acetothermus autotrophicum* | 262 |

| 7 | 504833703 | 18.02322528 | 4.171785302 | K0IMG9 | Ammonia monooxygenase/methane monooxygenase, subunit C | amoC2 Ngar_c33570 | *Nitrososphaera gargensis (strain Ga9.2)* | 187 |
| --- | --- | --- | --- | --- | --- | --- | --- | --- |
| 8 | 499631181 | 6.5 | 2.700439718 | Q3SJ14 | Nitrate reductase, alpha subunit (EC 1.7.99.4) | Tbd_1403 | *Thiobacillus denitrificans (strain ATCC 25259)* | 1252 |
| 9 | 503015657 | 6.5 | 2.700439718 | D8P8I1 | UDP-glucose 6-dehydrogenase (EC 1.1.1.22) | udg NIDE4145 | *Candidatus Nitrospira defluvii* | 439 |
| 10 | 503012584 | 3.25 | 1.700439718 | D8PBX5 | Pyruvate:ferredoxin oxidoreductase delta subunit (EC 1.2.7.1) | porD NIDE0972 | *Candidatus Nitrospira defluvii* | 211 |
| 11 | 499631181 | 6.5 | 2.700439718 | Q3SJ14 | Nitrate reductase, alpha subunit (EC 1.7.99.4) | Tbd_1403 | *Thiobacillus denitrificans (strain ATCC 25259)* | 1252 |
| 12 | 557791680 | 4.333333333 | 2.115477217 | U6BLN7 | Nitrite oxidoreductase beta subunit (Fragment) | nxrB | *Nitrospira moscoviensis* | 401 |
| 13 | 645910667 | 3.25 | 1.700439718 | A0A060PUI6 | Nitrite reductase (Fragment) | nirKI | *uncultured bacterium* | 149 |
| 14 | 499739558 | 3.25 | 1.700439718 | B8JDZ6 | Cold-shock DNA-binding domain protein | A2cp1_2724 | *Anaeromyxobacter dehalogenans (strain 2CP-1 / ATCC BAA-258)* | 66 |
| 15 | 503012632 | 2.166666667 | 1.115477217 | D8PC23 | Bacterioferritin (EC 1.16.3.1) | bfrB NIDE1021 | *Candidatus Nitrospira defluvii* | 158 |
